# The relative contributions of traffic and non-traffic sources in ultrafine particle formations in Tehran mega city

**DOI:** 10.1038/s41598-023-49444-z

**Published:** 2024-05-06

**Authors:** Farzaneh Jafarigol, Somayeh Yousefi, Ali Darvishi Omrani, Yousef Rashidi, Giorgio Buonanno, Luca Stabile, Sergei Sabanov, Mehdi Amouei Torkmahalleh

**Affiliations:** 1 Department of Chemical and Materials Engineering, School of Engineering and Digital Sciences, Nazarbayev University, Astana, Kazakhstan; 2 Department of Environmental Technologies, Environmental Sciences Research Institute, Shahid Beheshti University, Tehran, Iran; 3 Independent Researcher, Sari, Mazandaran 48197 Iran; 4 Department of Civil and Mechanical Engineering, University of Cassino and Southern Lazio, Cassino, Italy; 5 International Laboratory for Air Quality and Health, Queensland University of Technology, Brisbane, Australia; 6 Department of Mining Engineering, School of Mining and Geosciences, Nazarbayev University, Astana, Kazakhstan; 7 Division of Environmental and Occupational Health Sciences, School of Public Health, University of Illinois at Chicago, Chicago, IL 60612 USA

**Keywords:** Environmental sciences, Environmental impact

## Abstract

Emissions of ultrafine particles (UFPs; diameter < 100 nm) are strongly associated with traffic-related emissions and are a growing global concern in urban environments. The aim of this study was to investigate the variations of particle number concentration (PNC) with a diameter > 10 nm at nine stations and understand the major sources of UFP_s_ (primary vs. secondary) in Tehran megacity. The study was carried out in Tehran in 2020. NOx and PNC were reported from a total of nine urban site locations in Tehran and BC concentrations were examined at two monitoring stations. Data from all stations showed diurnal changes with peak morning and evening rush hours. The hourly PNC was correlated with NOx. PNCs in Tehran were higher compared to those of many cities reported in the literature. The highest concentrations were at District 19 station (traffic) and the lowest was at Punak station (residential) such that the average PNC varied from 8.4 × 10^3^ to 5.7 × 10^4^ cm^−3^. In Ray and Sharif stations, the average contributions of primary and secondary sources of PNC were 67 and 33%, respectively. Overall, we conclude that a decrease in primary emission leads to a decrease in the total concentration of aerosols, despite an increase in the formation of new particles by photo nucleation.

## Introduction

Particulate matter (PM) and nitrogen dioxide (NO_2_) are the two main urban pollutants emitted from various sources^1^. These pollutants are markers of traffic emissions in urban environments and their concentrations have declined in recent decades in developed and high-income cities around the world^2,3^. NO_2_ is known to have adverse effects on human health and vegetation, e.g., epiphytic lichens^4^. High concentrations of traffic-related pollutants such as particle number concentrations (PNC), nitric oxide (NO), and NO_2_ have been recorded around major roads^5,6^. Respiratory and cardiovascular adverse effects of these pollutants on populations living in near-road environments have been demonstrated through toxicology and epidemiology studies^7,8^.

PM is a complex mixture emitted from different sources that are available in the atmosphere at various sizes and is produced through several atmospheric processes^9^. Particles smaller than 100 nm are referred to as ultrafine particles (UFPs), especially in areas where geographical conditions reduce natural ventilation^10,11^. In general, in urban environments, PNC or UFP is dominated by particulate matter from traffic exhaust emissions, especially those from diesel engines^12–14^.

The impact of exposure to UFP_s_ on health has driven aerosol research in recent years. Studies have shown that UFPs disproportionately cause oxidative stress in cells^15^, and are more toxic than larger particles of the same composition due to the large surface area available for biological interactions with lung cells^16^. Time series epidemiological studies have shown that the number of particles and mass of particles predicts different health outcomes^13,17^. Several studies have shown that exposure to UFP related to traffic or indoor environment pollution is associated with cardiovascular effects as well as neurodegenerative diseases such as stroke, Alzheimer's disease, and Parkinson's disease^18–22^.

Efforts to mitigate the adverse health effects of particulate matter should be based on an understanding of key controlling factors, such as PNC or particle surface area concentration (PSC), rather than solely focusing on PM mass concentrations. Air quality standards have not been set for UFPs, and therefore, no efforts are being made to reduce their concentrations in the environment^23^. During the last decade, a number of experimental and numerical studies have improved the understanding of the release, dispersion, formation, exposure and health effects of UFPs. Only a handful of these studies have been conducted in emerging Asian cities where the majority of the world's urban population lives, and mostly in European cities^24^. The main reason for the lack of attention in Asian cities is their focus on compliance with the regulations on major pollutants, whose concentrations often exceed the standard^25^.

Since UFPs and NOx are derived from similar combustion processes (in automobile engines), UFP and NOx concentrations have been reported to be fairly correlated^26^. In urban areas, the origin of UFP_s_ may be primary or secondary^27^ and from the in situ, urban or regional nucleation^28,29^. In addition, a collocated monitoring of ambient BC and PNC can be a proper tool for assessment of the exposure to traffic emissions on roads^30,31^.

Various cities in distinct regions with different climatic conditions have undergone investigations to measure PNCs. For instance, in a traffic-influenced background site in Augsburg, elevated PNC was observed during late spring^32^. This increase in PNC was attributed to the prevailing southwest wind, facilitating particle transport from the main road to Augsburg. In an urban background station in Barcelona, the daily PNC chart showed that the concentrations with three high peaks are from 07:00 to 21:00. The morning and night peaks of PNC coincided with the intense vehicle activity in the area, while the noon peak could be attributed to new particle formation, which coincided with the highest solar radiation activity^33^. PNC values in the Helsinki metropolitan area were mainly due to the emission of local traffic exhaust gases and greenhouse gases from wood burning during the winter^34^. In London^28^, it was found that PNC is associated with BC and NOx. High PNC_s_ were observed in early spring/autumn and low concentrations in early summer/winter due to air masses coming from the mainland of Europe carrying particles. Daily charts for PNC in Shanghai showed that the peak of PNC coincided with traffic. This parameter also decreased in the afternoon around 12:00 to 16:00, which is associated with daily changes in mixing layer height (MLH) and source emissions^35^.

A study in Toronto^36^ found that PNC concentrations were low in spring and high in winter, a trend consistent with previous studies. Combustion sources (industrial and transportation) and secondary aerosols both originated in the south and southwest parts of Toronto and were the main sources identified. In 2010, a study on PNC ranging from 0.3 to 20 μm in the west-central parts of Tehran during two consecutive warm and cold seasons was conducted^37^. The particles collected from the five stations were simultaneously analysed in terms of mass and number by a laser-based Grimm dust monitor. The device used in this study measures particles of > 300 nm, and therefore, a large number of particles including the UFPs were excluded. Thus, the concentrations and sources of UFPs in Tehran have remained unknown so far and require investigations.

Tehran is the capital of Iran and a mega city surrounded by mountains with a height of about 3800–1000 m in the north, south, and east, which intensifies pollution in the city. The population of this city is highly exposed to air pollution, especially PM^38^. Additional factors such as rapid urbanization, uncontrolled vehicle emissions, and lack of infrastructure have reduced Tehran's air quality^39^. Vehicles are considered to be the main cause of air pollution in Tehran. About 2 million cars over the age of 20 travel daily and emit large amounts of PM^40^. Tehran, like other mega cities, encounter important challenges related to air quality. Municipality buses, passenger cars, and trucks constitute the three main categories of Tehran's transportation fleet during the morning rush traffic hour. Notably, within this timeframe, which falls within the restrictions imposed on heavy-duty diesel vehicles, trucks are absent from the vicinity of the measuring stations^41^. The primary air pollutants in Tehran include PM_10_, SO_2_, NO_2_, HC, O_3_, and CO, and mobile pollution sources account for 80–85% of their emissions^42^. Previous studies have identified sources of vehicle pollutants, secondary aerosols, and industrial emissions as the predominant sources of PM in Tehran with minimal contributions from road dust, biomass burning, oil combustion, and soil^43^. However, thus far, no study addressed the concentrations of UFPs in Tehran and clarified their potential sources.

This study aims to explore the fluctuations in PNC within the size range of 10–300 nm across nine stations in Tehran. The primary objective was to discern the major sources of UFPs, distinguishing between primary and secondary origins in the city. The correlation between PNC and criteria air pollutants was examined. Ultimately, the investigation sought to pinpoint the influence of local sources and meteorological parameters on the dispersion of UFPs in Tehran.

## Methods and materials

### Study area and monitoring sites

With an area of 700 square kilometres, Tehran extends from latitude 35° 35′ N to 35° 48′ N and longitude 51° 17′ E to 51° 33′ E. It is located at more than 1200 m above sea level with a slope of 700 m between the highest and lowest points. Tehran has approximately 13.3 million residents and 10 million commuters^44^. It is located in the foothills of the Alborz Mountains in the north, Jajrood valleys in the east, Karaj valleys in the west, and the south western margin of the central desert from the south. Due to the prevailing meteorological conditions and topography of Tehran, stable meteorological conditions and temperature inversion occur more frequently in winter and autumn, which is one of the main reasons for severe air pollution^45^. However, man-made factors such as rapid population expansion, rapid conversion of agricultural land and natural objects into industrial sites and urban areas, and a relatively old vehicle fleet contribute significantly to the severity of air pollution in Tehran.

### Data collection

**Figure 1:**
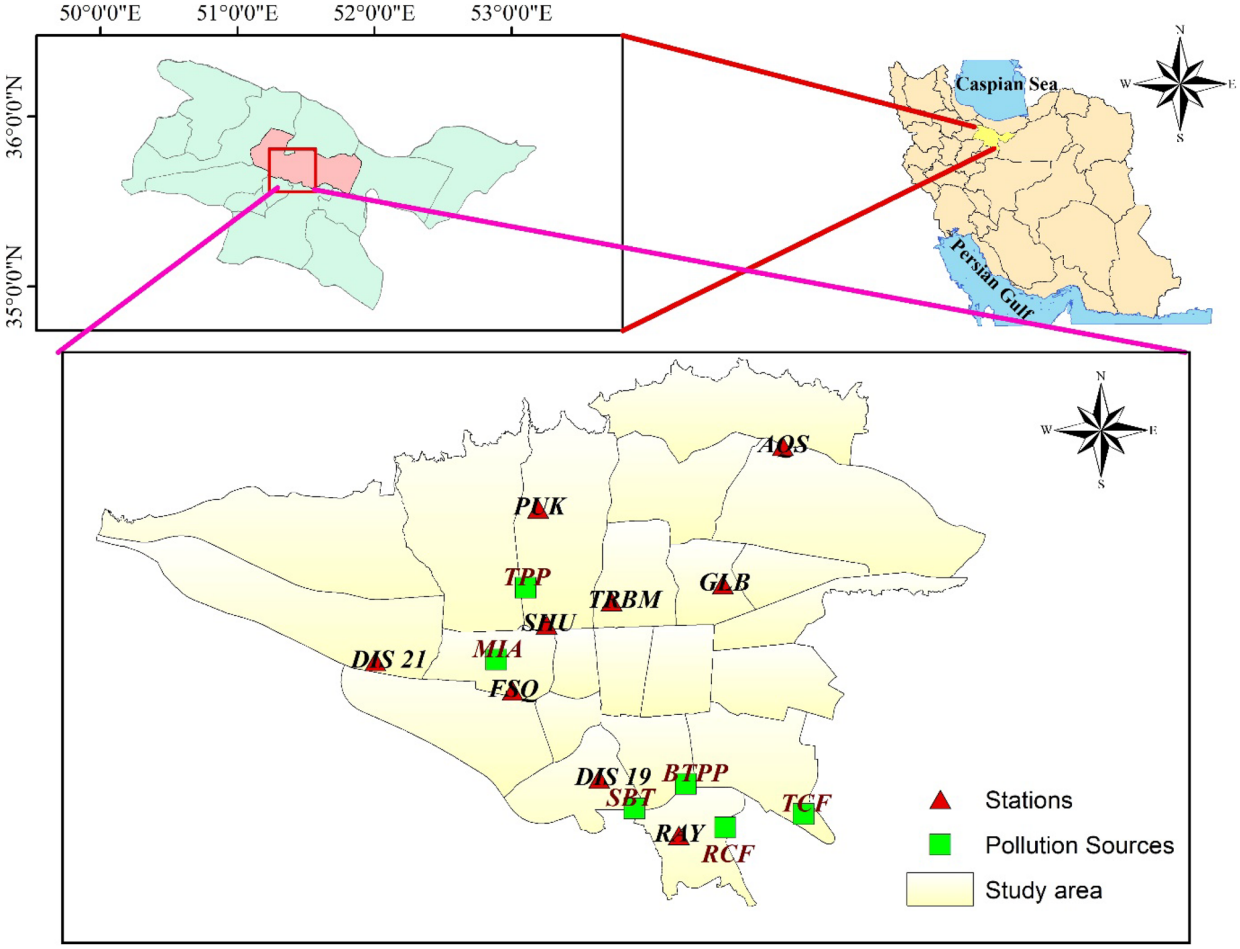
Locations of nine monitoring stations, main point sources which may contribute to PNC, and the major traffic roads around the stations, including Mehrabad airport (MIA), Tarasht power plant (TPP), B`esat thermal power plant (BTPP), South Bus Terminal (SBT), Ray and Tehran cement factories (RCF, and TCF).

Hourly concentrations of NOx in 2020 were obtained from nine air quality monitoring stations (Table 1 and Fig. 1) operated by Tehran's Air Quality Control Company (AQCC) (http://air.tehran.ir/). Hourly BC data at two air quality monitoring stations (SHU and RAY) were obtained from Tehran Air Quality Control Company. The Environnement S.A-AC32M analyzer was used to monitor NOx concentrations. Criteria air pollutants, including CO, SO_2_, NOx, PM_2.5_, and O_3_ were measured in 1-h time resolution. The BC concentrations were measured in 1-min time resolution by using an AE33 BC monitor manufactured by Magee Scientific, USA. PN 8060 type filter tapes with a sensitivity parameter (C) of 1.39 and leakage parameter (Z) of 0.01 were employed in AE33 Aethalometers. Quality assurance/quality control (QA/QC) procedures were conducted in accordance with BS EN 12,341:2014 and BS EN 14,626:2012 for PM and CO analyzers, respectively. QA/QC procedures for Aethalometers were performed following the official user manual of the analyzers published by Magee Scientific^41^. The particle number concentrations were measured using a NanoTracer, Aerasense (Netherlands). This device is able to determine the average particle size and the particle number concentration in the size range of 10–300 nm up to 10^6^ cm^−3^. PNC sampling was performed at nine stations such that at each station, NanoTracer was operated for six consecutive days 24 h a day. The sampling intervals were every 10 s; then; the corresponded data converted to 1-h averages. The NanoTracer employed in the experimental studies underwent calibration conducted by the manufacturer. Since the NanoTracer is not a reference instrument, a correction factor has to be applied to its readings. The correction factor was obtained through collocated measurements of a NanoTracer and a reference instrument such as SMPS, FMPS or CPC. The ratio between the concentrations recorded by NanoTracer and a reference instrument is defined as correction factor. The PNC data in this study were adjusted using the correction factor reported in reference^46^. The average correction factor applied for NanoTracer was determined to be 1.9 ± 0.3.

**Table 1:** Main details of the selected monitoring stations.

Station name	Type of station	coordinates		Date
		Lat	Long	
Aqdasiyeh (AQS)	Urban-Residential	35.79	51.48	2020/04/12–2020/04/18
Fath Square (FSQ)	Urban-Traffic	35.68	51.34	2020/02/12–2020/02/18
Ray (RAY)	Urban-Industrial	35.60	51.42	2020/03/17–2020/03/23
Golbarg (GLB)	Urban- Residential	35.73	51.50	2020/03/30–2020/04/06
Punak (PUK)	Urban- Residential	35.76	51.33	2020/04/18–2020/04/23
Sharif University (SHU)	Urban-Traffic	35.70	51.35	2020/01/22–2020/01/27
Tarbiat Modarres University (TRBM)	Urban-Traffic	35.72	51.38	2020/01/27–2020/02/01
District 21 (DIS 21)	Urban-Traffic	35.70	51.24	2020/02/07–2020/02/12
District 19 (DIS 19)	Urban-Traffic	35.63	51.36	2020/03/11–2020/03/17

### The Segregation of the primary and secondary sources of PNC

Equations (1) and (2) outline the methodology employed in this study to estimate the contribution of primary and secondary particles to the total PNC^47^. During morning rush hours (primarily 6–9 a.m.), a linear regression correlation is established between PNC and BC, and the estimated PNC derived from this equation is denoted as N_1_. Subsequently, this estimating equation is applied throughout the remainder of the day, utilizing measured BC concentrations to estimate N_1_. N_2_ is then calculated by subtracting N_1_ from the measured total PNC (N) over the course of the day. In this approach^47^, N_1_ represents primary traffic emissions, while N_2_ encompasses various scenarios, including newly formed particles in the atmosphere from gas precursors, low BC-bearing primary particles from diverse urban sources excluding traffic, and particles transported by air masses^14,48,49^. S_1_ (particles/ng BC) denotes the slope of the correlation between N and BC during the morning rush hours. N represents the field-measured total number concentration, and BC represents the field-measured black carbon concentration. This methodology has been successfully applied in prior studies conducted in European cities^50,51^, as well as in an Asian megacity and boreal forest site in Finland^52^.

1 $${\text{N}}_{1} = {\text{ S}}_{1} \times {\text{ BC}}$$

2 $${\text{N}}_{2} = {\text{ N }} - {\text{ N}}_{1}$$

Reference^14^ considered the first percentile of the N/BC ratio during the morning rush hour to develop a correlation between N and BC while we used all morning rush hour data since our dataset was not as large as that used by the reference^14^.

### Source identification using conditional bivariate polar function (CBPF)

The CBPF method^53^ was used to identify potential PNC emission sources. CBPF analysis can identify potential sources around stations and estimate the likelihood that high concentrations will occur there. The CPF method^54^, which incorporates wind speed (or any other parameter) as a third variable. Using the ordinary CPF, we can estimate how likely it is that a pollutant concentration measured in one wind sector will exceed a certain threshold. Unlike wind direction sectors alone, CBPF, defined as Eq. (3), takes into account different wind direction and speed ranges:

3 $${CBPF}_{\Delta \theta ,\Delta u}=\frac{{m}_{\Delta \theta ,\Delta u|C\ge x}}{{n}_{\Delta \theta ,\Delta u}}$$

As ${m}_{\Delta \theta ,\Delta u}$ represents the number of samples taken in a given wind sector $\Delta \theta$ at wind speeds $\Delta u$, C represents a pollutant concentration, x indicates a high percentile of concentration, such as 75th, and ${n}_{\Delta \theta ,\Delta u}$ indicates the total number of samples taken during the wind direction-speed interval. In the R language (version 4.3.0^55^), we performed these analyses utilizing the "OpenAir" package^56^.

### Ethical Responsibilities of Authors

All authors have read, understood, and have complied as applicable with the statement on "Ethical responsibilities of Authors" as found in the Instructions for Authors. This study does not involve human subjects or animals.

## Results and discussion

### Spatial and temporal variations of PNC and NOx concentrations

**Figure 2:**
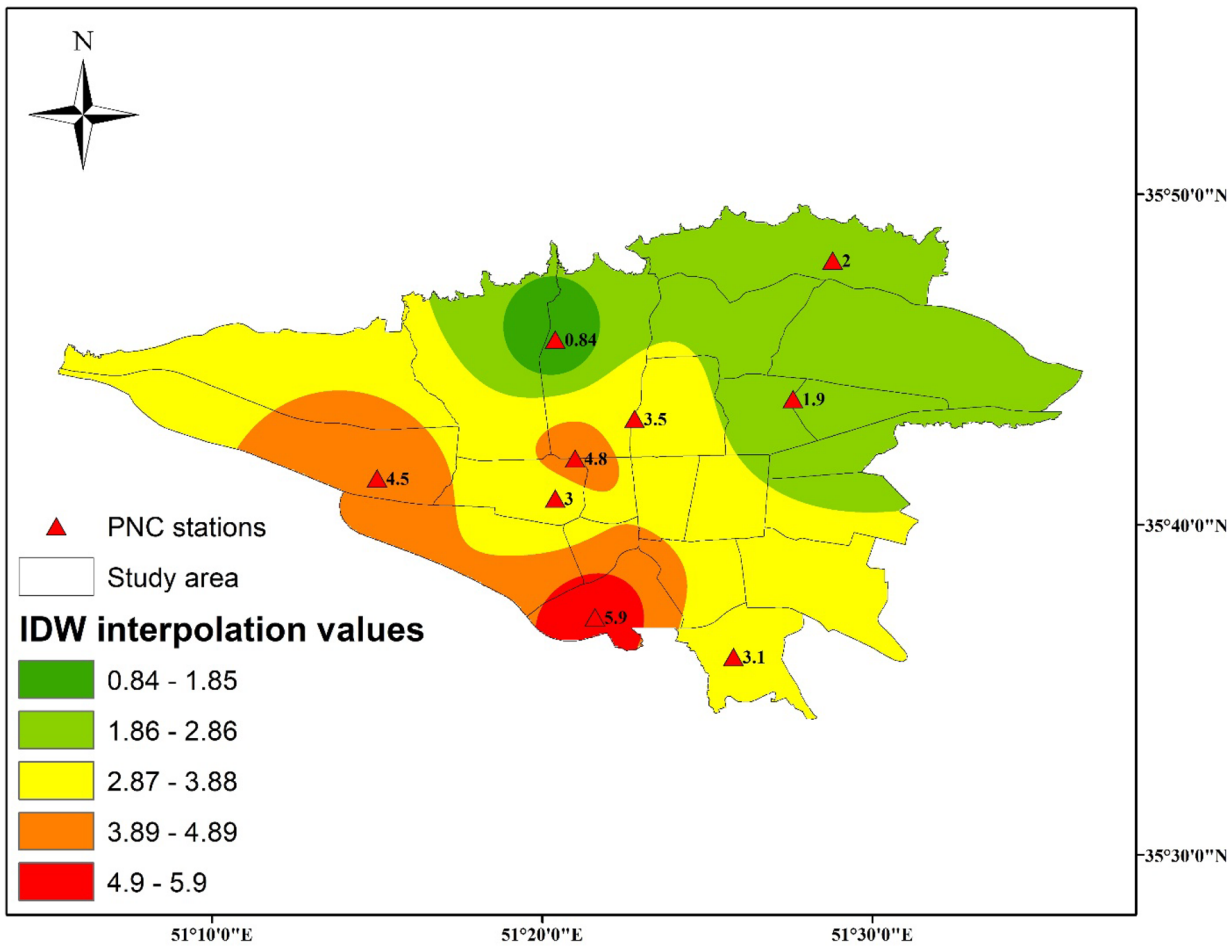
Total values of PNC (× 10^4^ particle/cm^3^) at all stations during the measurement periods with the corresponding IDW interpolation values.

Figure 2 illustrates the overall mean PNC values at nine monitoring stations over the study period. Among these, the lowest PNC value (0.84 × 10^4^ particle/cm^3^) was registered for the residential station PUK, while urban-traffic stations DIS19, followed by SHU, recorded the highest values (5.9 and 4.8 × 10^4^ particle/cm^3^, respectively). Utilizing inverse distance-weighted interpolation, the total PNC values demonstrated a spatial pattern with an increase from northern to southern city areas. This pattern suggests the influence of topography and mixed layer height, with rougher and higher conditions in the northern regions aiding in the dispersion and dilution of PNC. Conversely, the central and southern areas, characterized by a higher concentration of pollution sources, including direct emissions from road traffic and industrial zones, experienced elevated PNC values.

**Figure 3:**
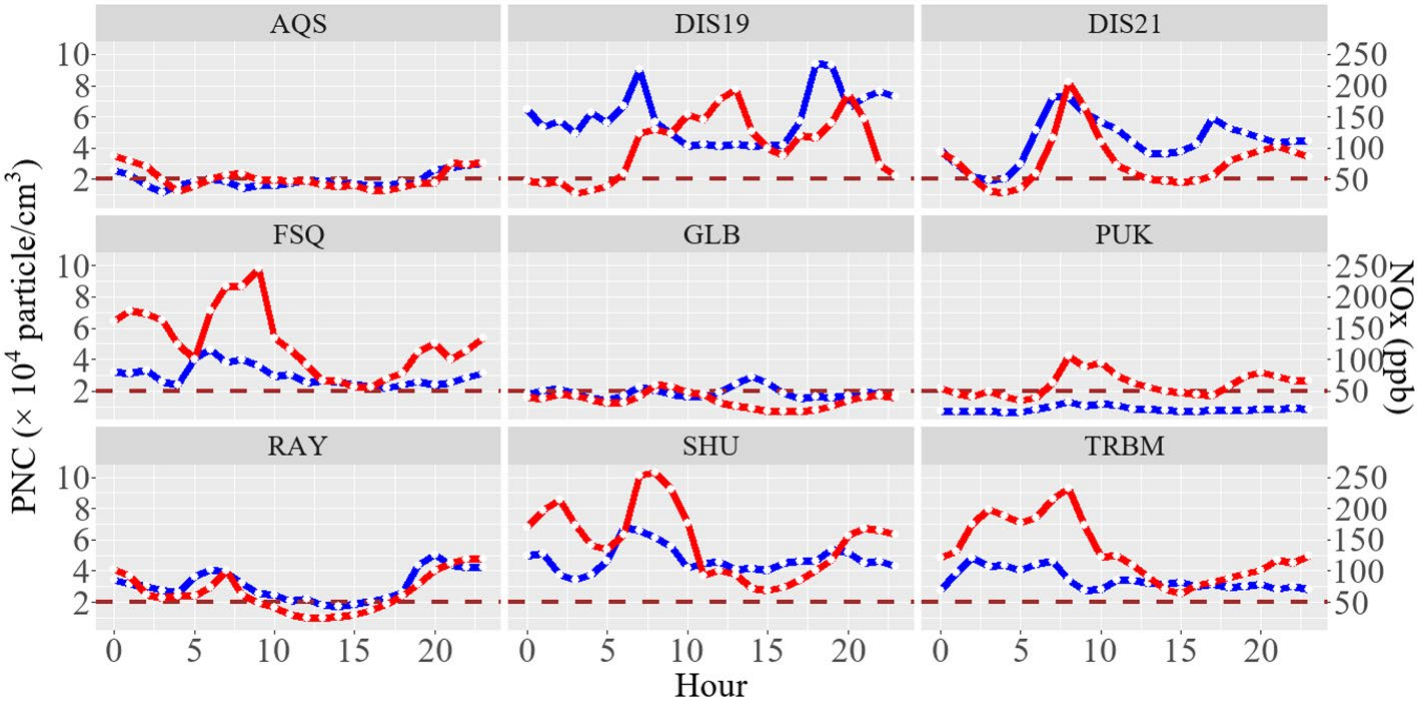
Diurnal average concentrations of PNC (blue, left side), and NOx (red, right side) at all stations. The brown dashed line represents the WHO high value threshold for UFP concentration for 1-h average.

Figure 3 shows the diurnal patterns of PNC (left side) and NOx (right side) across various stations throughout the sampling period. Stations PUK, GLB, and AQS consistently maintained PNC values close to the WHO high value threshold for UFP concentration (2 × 10^4^ particle/cm^3^ for a 1-h period) for most hours of the day^57^. However, other stations consistently exceeded this limit throughout the entire day. DIS19, DIS21, Ray, and SHU exhibited bimodal peaks, with PNC rising from 5 (local time) and peaking between 6 to 8, followed by a second peak in the afternoon from 17 to 20 (local time). Conversely, FSQ, TRBM, and AQS experienced unimodal peak values in the early hours, predominantly between 0 and 5 a.m. These dual increases in PNC are likely associated with morning and evening rush hours, as well as the influence of meteorological condition. In Tehran, traffic regulations impose restrictions on heavy-duty diesel vehicles (HDDVs) during daytime hours. Specifically, heavy-duty trucks are permitted within the city from late night to early morning on workdays (Saturday to Wednesday) and from midnight to early morning on weekends (Thursday and Friday). Other diesel vehicles, including light delivery trucks and public transportation, have the flexibility to operate in Tehran almost continuously^41^. The PNC experienced a marked increase, specifically at DIS19, DIS21, FSQ, SHU, and RAY, and sustained high levels when HDDVs were granted access to the streets, particularly when the MLH was low. As the boundary layer height increased, and heavy trucks were prohibited, the PNC concentration experienced a significant decrease, maintaining a lower level throughout the day until night-time.

Given their proximity to traffic sources, stations DIS21, RAY, SHU, and TRBM exhibited similar trends in both NOx and PNC, displaying simultaneous peaks and troughs. This consistency suggests insufficient time for pollutants to mix within the MLH, indicating that PNCs emitted directly from vehicles started to increase or decrease at these stations almost concurrently with NOx variations. The NOx concentration profile distinctly shows two peaks: one during night-time for HDDVs traffic and another during the daytime for the morning rush hour of light-duty vehicle (LDV) traffic. Notably, the profile underscores the pronounced impact of LDVs on NOx concentration, contrasting with the comparatively lower effect of HDDV traffic during night-time. NOx concentrations ranged from 25.4 to 293 ppb at urban-traffic stations and 17.2–105 ppb at urban-residential stations. Furthermore, NOx concentrations were generally lower in the afternoon than in the morning at most stations, indicating the dominant influence of traffic emissions in the morning. Stations located closer to the center of Tehran, such as SHU, TRBM, and DIS21, exhibited higher NOx concentrations due to increased traffic load and elevated levels of domestic and commercial activities.

### Contribution of primary and secondary sources in PNC

Table 2 presents the average percentage of N_1_ and N_2_ using hourly concentration data for RAY and SHU stations. In urban environments with traffic emissions, an observed association between BC concentration and PNC has been reported^10,58,59^. Scattered plots of BC versus N were analyzed for traffic rush hours in the morning, resulting in estimated values of S_1_ (expressed as particles/ng BC) at 14.4 × 10^6^ and 15.9 × 10^6^ for RAY and SHU stations, respectively. Throughout the day at RAY station, the contribution of N_1_ to PNC was generally higher than that of N_2_, except during the noon-afternoon time when the proportions were 34.4% for N_1_ and 65.6% for N_2_. This observation underscores the significant contribution of primary particles associated with traffic during those specific hours. At SHU station, the contribution of N_1_ was lower from noon till evening, accounting for 42.5% compared to 57.5% for N_2_. N_2_ reached peak levels during midday, constituting 65.6% and 51.5% at RAY and SHU, respectively. This peak coincides with the anticipated maximum of photochemical nucleation, attributed to the photo-oxidation of gaseous precursors in the atmosphere during periods of maximum solar radiation. The highest contribution of N_1_ was observed at RAY during night-time and morning rush hours due to its location far from the city's core, being influenced by mixed traffic and industrial sources. Additionally, RAY station is situated near Tehran's ring road, where HDDVs are permitted to pass without time restrictions. Consequently, increased heavy diesel vehicle traffic during the night resulted in higher emissions of PNC compared to daytime^41^. Particles below 100 nm, which frequently dominate urban PNC, are directly emitted into the atmosphere from combustion processes associated with industry, traffic, domestic heating, and other sources such as vehicle brakes. Emissions from vehicles can contribute to the presence of both primary and secondary particles in the atmosphere. These pollution episodes may be mitigated through taking a wide variety of implementations, such that the implementation of traffic restrictions, particularly in central areas like The Odd–Even Traffic Rationing zone, The Restricted Traffic Zone, and The Low-Emission Zone, resulted in reduced emissions of CO, NOx, VOCs, and SOx by 4.5%, 2.9%, 5.8%, and 2.7%, respectively^41^.

**Table 2:** Total average percentages of N1 and N2 on an hourly basis during the day.

Time	Duration	RAY		SHU	
		N1 (%)	N2 (%)	N1 (%)	N2 (%)
Night	0-5h	91.3	8.7	78.4	21.6
Morning	7–8 h	93.5	6.5	71.0	29.0
Noon-afternoon	11–14 h	34.4	65.6	50.3	51.5
Evening	17–19 h	74.8	25.2	42.5	57.5

### Correlations between PNC and criteria air pollutants

**Figure 4:**
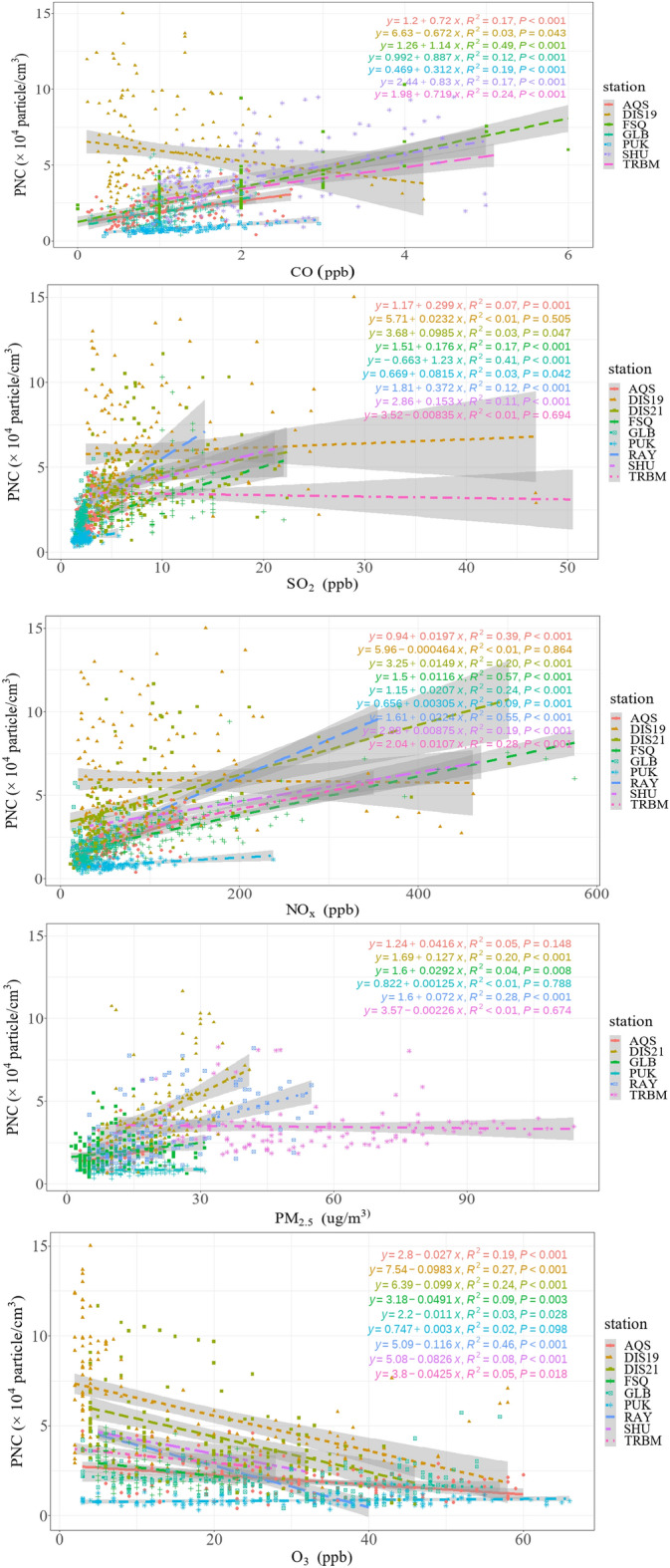
Correlations between hourly average PNC and criteria air pollutants with 0.95 confidence interval at nine stations.

The relationships between hourly PNC and concentrations of CO, SO_2_, NOx, PM_2.5_, and O_3_ were examined through a single-variable regression method, as depicted in Fig. 4. The results revealed moderately low but significant correlations (p-value ≤ 0.05) between PNC and CO for FSQ (R^2^ = 0.49) and between PNC and SO_2_ for GLB (R^2^ = 0.41). For all other stations, the correlations were low, with R^2^ values ranging between 0.17 and 0.24 for CO and between 0.01 and 0.17 for SO_2_. Conversely, notable correlations, ranging from relative-high to moderate-low, were observed with NOx at FSQ and RAY (R^2^ = 0.57 and 0.55, respectively), as well as AQS (R^2^ = 0.39), DIS21 (R^2^ = 0.20), GLB (R^2^ = 0.24), and TRBM (R^2^ = 0.28). It is noteworthy that similar patterns in the relationship between PNC and NOx were observed in studies conducted at surface stations in Gothenburg^26^, London^60^, and Stockholm^61^, reinforcing the consistency of our findings with existing research. This pattern can be attributed to the fact that a substantial proportion of urban NOx emissions is associated with diesel vehicles^62,63^. Despite comprising only 2.4% of Tehran's vehicle fleet, diesel vehicles contribute significantly, accounting for more than 41%, 64%, and 85% of the NOx, SOx, and PM emissions, respectively^41^. Moderate-low to moderate-high correlations between PNC and NO and NO_2_ were similarly reported for both urban (R^2^ = 0.27 and 0.35, respectively) and traffic-oriented stations (R^2^ = 0.70 and 0.63 for NO and NO_2_, respectively) across European countries^64^. The mean PNC values and R^2^ values for PNC-NOx correlations from other studies are detailed in Table 3, demonstrating consistently elevated levels in metropolitan areas and proximity to highways. Notably, strong correlations between particle number concentrations and NOx were observed in most studies. Marylebone Road recorded the highest PNC among the locations listed in Table 3, situated alongside a road with a traffic flow exceeding 80,000 vehicles per day within a street valley^65^. While Tehran's PNC exceeded values in all the cities mentioned in Table 3, it only fell below Hornsgatan and Marylebone Rd. Discrepancies in PNC among cities may arise not only from differing source profiles but also from variations in the instrumentation used for measurement, especially considering the potential impact of lower cut sizes on total measured PNC. For PNC and PM_2.5_, correlations were either insignificant (e.g., AQS, PUK, and TRBM) or significantly moderate-low at DIS21 and RAY (R^2^ = 0.20 and 0.28, respectively). Global study^66^, including ten cities across North America, Europe, Asia, and Australia, also reported low correlations of PM_2.5_ and PNC (R^2^ = 0.01 to 0.48). Thus, PNC and PM_2.5_ measurements do not represent each other adequately, highlighting the need for more precise pollutant indicators such as PNC or BC mass^41^ instead of total PM_2.5_ mass for more effective policy implementation. Finally, negative and very low correlations for O_3_ with PNC were observed at FSQ, GLB, SHU, and TRBM (R^2^ < 0.1). In contrast, notably higher PNC and O_3_ correlations were identified at DIS21, DIS19, and RAY (R^2^ = 0.24, 0.27, and 0.46, respectively). The negative slopes for the former group indicate that O_3_-rich sources of PNC emissions are not significant in Tehran, at least during the measurement period in this study.

**Table 3:** Particle Number Concentrations and R^2^ values for PNC-NO_*x*_ reported in other studies.

Location	Country	Author	Site description	Mean concentration (cm^-3^)	R^2^
Vienna, Linz, Graz	Austria	^67^	Urban	29,300–31,100	–
				16,200–20,600	–
Birmingham	UK	^13^	Background	28,600–36,600	0.20
Vilnius	Lithuania	^68^	Urban Background	8000–10,000	–
Hope St.eSt.,	Glasgow	^65^	Kerbside/street canyon	23,564	0.79
Enoch Sq			Urban centre	12,851	
Montrose St			Background/street canyon	11,095	
Marylebone Rd	London		Street canyon	109,953	0.78
North Kensington			Urban background	23,407	
Hornsgatan	Stockholm	^61^	Wet and dry road surface condition	70,000	0.14
Gothenburg	Sweden	^26^	Urban background	4,000–10,000	0.16
					0.55
Barcelona	Spain	^69^	Urban background	2,000–8,000	–
Montseny			Regional background		
Amsterdam	Netherlands	^28^	Urban Background	1552	0.086
Antwerp	Belgium			1709	0.6
Leicester	UK			1541	0.58
London	UK			1007	0.51
Barcelona	Spain	^14^	Urban Background	12,607.7	0.7
Huelva			Urban industrial	16,751.8	0.3
Tenerife			Urban Background	14,150.5	0.6

### CBPF analysis results

**Figure 5:**
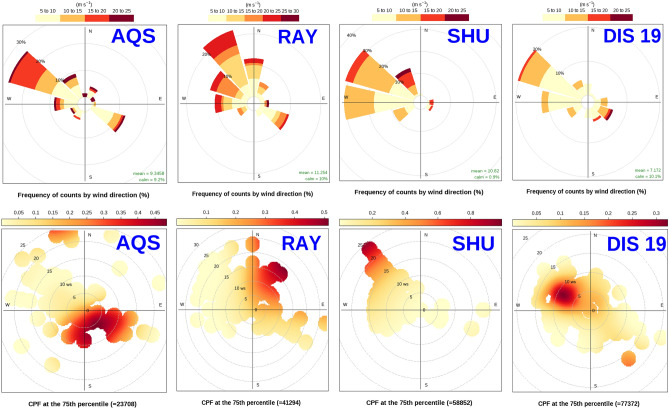
Dominant wind directions with CBPF polar plots analysis showing how contributions of different distant and local sources are affected by wind direction and wind speed.

In Fig. 5, we showed the dominant directions of wind and the CBPF analysis for PNC located in the north (AQS, residential), center (SHU, and DIS19, traffic), and south (RAY, traffic-industrial), where only the 75^th^ percentile was used to distinguish the most important sources of pollution at each station. The traffic sources and domestic heating emissions at AQS, GLB, and DIS19 stations were predominantly local in nature since low wind speeds prevail. These stations showed 45%, 25%, and 30% probabilities coinciding with 2.4 × 10^4^, 2.3 × 10^4^, and 7.7 × 10^4^ particle/cm^3^ (75th percentiles) concentrations when the wind is from ESE, S, and WNW directions and the wind speed is in the range of 5–10 m/s, respectively. The above-mentioned directions contain a number of major roads that could have an impact on traffic-related sources. For instance, Sadr and Sayyad Shirazi highways are located 1.5 km away from AQS, Baqeri Expressways are located 0.5 km away from GLB, Sa'idi and Kazemi Expressways are located just 0.5 km away from DIS19. Several sources of pollution have been identified in the NW of DIS21, which could be attributed to industrial complexes (food and automotive industries), as well as the Lashkari Expressway. Despite the fact that the Azadegan Expressway is located in the SE of this station, there is more than a 30% probability that wind speed ranges between 20 and 30 m/s and winds from the NW could significantly increase particle concentrations to 5.3 × 10^4^ particle/cm^3^, highlighting the importance of meteorological impacts on pollutant long-range transport and dispersion. Similarly, in spite of SHU station’s proximity to the Mehrabad airport, however; particle concentrations are more than 80% probable to be originated by NW wind directions and 20–30 m/s wind speed to be 5.8 × 10^4^ particle/cm^3^, which are the locations of the Tarasht power plant and the Nuri Expressway. FSQ station, located just close to the airport and residential environment, is primarily affected by domestic sources such as natural gas, liquid petroleum, and propane gas which are used for building spaces heating or emitted from kitchens during cooking, as well as a high rate of emission from the airport. Airports are responsible for emitted pollutants such as PN, PM_2.5_, and black carbon; PN concentrations at airport sites were approximately four times greater compared to the freeway^70^. The W wind component and 30–40 m/s wind speed highlight the impacts of the Fath highway aside from the sources mentioned above, which can significantly increase particle concentrations probabilities by more than 25% and 15% to be 3.2 × 10^4^ particle/cm^3^, respectively. Similar to FSQ, PUK and TRBM stations also suffer from domestic sources from SW and SE with wind speeds between 15 and 25 m/s, respectively, which contribute to only 9.2 × 10^3^ particle/cm^3^ by 60–80% and 3.7 × 10^4^ particle/cm^3^ by more than 80% probabilities, respectively. Because the strongest wind direction comes from the W-NW directions at both stations, and the 75^th^ percentile at PUK station is considerably lower than other stations, the role of the major road, Ashrafi Esfahani Expressway, located in SE direction of PUK station, cannot be discussed precisely in terms of long-range transport contribution to pollution. Along with the vicinity of the Avini Expressway, RAY station is also affected by the Be'sat power plant. A particle concentration of 4.1 × 10^4^ particle/cm^3^ is more than 50% likely to result from N wind directions with a speed of 15–20 m/s at this urban traffic station. As a result of distillate oil and natural gas usage in Iran during the cold and warm seasons, they emit high levels of NOx, SO_2_, PM, and greenhouse gases from stationary internal and external combustion^71^, which emphasize the significance of power plants studied above.

### Limitations of this study

It is important to highlight that in stations characterized by elevated BC concentrations, the calculated values of N_2_ appeared to be negative. This suggests that the application of the method proposed by^47^ may not be universally applicable under conditions with high BC concentrations. However, to draw more definitive conclusions on this matter, further investigation through a comprehensive study with a larger dataset is warranted. Moreover, a combination of particle size and number may shed light on the primary or secondary production of UFPs; thus, considering that particles with primary mode diameter peaks at 30–35 nm and 60–80 nm are linked to spark-ignition and diesel vehicle emissions, respectively^72^, conducting particle number size distribution analyse would be the future work of the present study. This additional step can enhance the ability to discern local or regional traffic sources, providing a more nuanced understanding of the contributors to particle number concentration.

## Conclusions

In Tehran, the primary source of PNC is predominantly linked to vehicle exhaust emissions, particularly heightened during rush hours. Secondary particle formation in the ambient air is observed mainly during noon or early afternoon. The diurnal PNC trend follows a pattern with peak values occurring during morning and evening rush hours. This study establishes a positive correlation between changes in urban PNC and BC as well as NOx. To differentiate between primary and secondary sources of PNC, the segregated method is applicable when BC and PNC are measured simultaneously at the same stations. In Tehran, specifically at RAY and SHU stations, the average contribution of primary and secondary sources to PNC was determined to be 67% and 33%, respectively. The CBPF analysis identified local traffic as the primary source of PNC emissions in Tehran. Additionally, the study underscored the influence of meteorological factors that may contribute to the transport of pollution over long distances from distant sources to the receptor. This highlights the significance of the MLH as a determining factor during the daytime in the cold season in Tehran. Notably, the study revealed that traffic regulations for HDDVs played a significant role in influencing PNC levels at traffic stations during the night-time. It was found that PNC, as a local pollutant, is directly impacted by the emissions from the diesel fleet, particularly heavy-duty trucks, indicating that HDDVs traffic stands as the main source of PNC emissions in Tehran. Consequently, the study suggests that phasing out old HDDVs and replacing them with newer technology vehicles could yield beneficial outcomes. The average PNC values observed in most stations in Tehran exceeded those in many cities reviewed in the study. In conclusion, the study recommends that reducing primary emissions in Tehran would be a practical approach to decrease the population's exposure to UFPs. Additionally, controlling the formation of new particles could also significantly contribute to reducing such exposure.

## Data Availability

Upon a reasonable request to the corresponding author of this study, the data generated and/or analysed during this study can be available.
