# Author Correction: The relative contributions of traffic and non-traffic sources in ultrafine particle formations in Tehran mega city

**DOI:** 10.1038/s41598-024-64500-y

**Published:** 2024-08-02

**Authors:** Farzaneh Jafarigol, Somayeh Yousefi, Ali Darvishi Omrani, Yousef Rashidi, Giorgio Buonanno, Luca Stabile, Sergei Sabanov, Mehdi Amouei Torkmahalleh

**Affiliations:** 1 Department of Chemical and Materials Engineering, School of Engineering and Digital Sciences, Nazarbayev University, Astana, Kazakhstan; 2 Department of Environmental Technologies, Environmental Sciences Research Institute, Shahid Beheshti University, Tehran, Iran; 3 Independent Researcher, Sari, 48197 Mazandaran Iran; 4 Department of Civil and Mechanical Engineering, University of Cassino and Southern Lazio, Cassino, Italy; 5 International Laboratory for Air Quality and Health, Queensland University of Technology, Brisbane, Australia; 6 Department of Mining Engineering, School of Mining and Geosciences, Nazarbayev University, Astana, Kazakhstan; 7 Division of Environmental and Occupational Health Sciences, School of Public Health, University of Illinois at Chicago, Chicago, IL 60612 USA

Correction to: *Scientific Reports* 10.1038/s41598-023-49444-z, published online 06 May 2024

The original version of this Article contained an error in the spelling of the author Luca Stabile which was incorrectly given as Luca Stabil.

The original Article has been corrected.

